# The fate of surplus laboratory animals: Minimizing the production of surplus animals has greatest potential to reduce the number of laboratory animals

**DOI:** 10.15252/embr.202256551

**Published:** 2023-01-30

**Authors:** Hartmut Wewetzer, Tobias Wagenknecht, Bettina Bert, Gilbert Schönfelder

**Affiliations:** ^1^ Department of Risk Communication German Federal Institute for Risk Assessment Berlin Germany; ^2^ German Centre for the Protection of Laboratory Animals (Bf3R) German Federal Institute for Risk Assessment Berlin Germany; ^3^ Charité – Universitätsmedizin Berlin, corporate member of Freie Universität Berlin and Humboldt‐Universität zu Berlin Berlin Germany

**Keywords:** Economics, Law & Politics, Methods & Resources, Pharmacology & Drug Discovery

## Abstract

To meet regulatory requirements and the political pressure to minimize the number of animals used in research, it is critical to reduce the production of surplus animals.

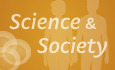

Modern medicine is not possible without animal testing. Whether it is the novel immunotherapies against cancer or the vaccines against SARS‐CoV2, biomedical research and drug development crucially depend on experiments and tests using animals (Genzel *et al*, 2020). The broad public acceptance of the use of animals in research is largely due to the fact that it has delivered medical innovations that have enormously improved human quality of life and life expectancy. But, this has come with a price of millions of ‘surplus’ animals that are not being used in experiments and therefore have to be killed. For the sake of improving animal welfare and for meeting the goals of the EU regulation on the use of animals in research, science needs to find ways to reduce the number of ‘surplus’ animals.

Even despite careful planning, breeding programs create many animals without the intended characteristics, that is, the correct genetic background.

Genetically modified animals are an indispensable tool to study the pathogenicity of human diseases and to model human physiology in drug testing. The most important animal model is the mouse, the generation and maintenance of which involve sophisticated breeding programs. Even despite careful planning, breeding programs create many animals without the intended characteristics, that is, the correct genetic background. These genetically ‘unsuitable’ laboratory animals often cannot be used for further experiments and are being killed. The ‘wrong’ sex or age are also causes that render laboratory animals unsuitable for experiments.

Many other surplus animals arise from scientific or commercial stock and maintenance breeding to enable animal testing in a timely manner. As many research projects are carried out under time pressure and/or with time‐limited funding, breeders maintain a large stock of animals with diverse genetic backgrounds so as to quickly satisfy demand or to generate the required genetic combinations for specific experiments. In addition, reviewers of a submitted research paper frequently ask for additional animal experiments from the authors. The only way to comply with the required revision within the limited timeframe is to obtain stock animals from commercial or institutional breeders. Stock animals that are not used for experiments are eventually culled. In this regard, both funding agencies and scientific journals could extend the deadlines for funding or for submitting revisions: breeders would have more time to generate the required number of animals on demand instead of having to keep a large stock in reserve.

## A hidden sacrifice

The absolute numbers of surplus animals produced globally remains unknown; there are only numbers available for Europe as the EU Directive 2010/63/EU on animal welfare (https://eur-lex.europa.eu/LexUriServ/LexUriServ.do?uri=OJ:L:2010:276:0033:0079:en:PDF) requires that the total numbers of animals created for and used in research in Europe is published every 5 years. In 2017, 12.6 million surplus animals were killed in the EU (https://eur-lex.europa.eu/legal-content/EN/TXT/HTML/?uri=CELEX:52020DC0015&from=EN). This number is significantly higher than the 9.4 million animals that were used in experiments (https://eur-lex.europa.eu/legal-content/EN/TXT/HTML/?uri=CELEX:52020DC0016&from=EN). Animal welfare organizations have long demanded that other major scientific players, such as the USA or China, should also make public how many laboratory animals they use each year.

Germany has gone a step further and now requires publishing these numbers each year from 2021 onward. The 2021 statistics lists 2.55 million surplus animals and 2.5 million animals used for scientific research (https://www.bf3r.de/de/verwendung_von_versuchstieren_im_jahr_2021‐309160.html). These figures highlight how many animals are actually needed for research and how many others remain in the shadow of public attention.

## Animals have an intrinsic value

During the past few decades, research has contributed significantly to raising awareness of animal welfare (Marchant‐Forde, 2015). The growth of scientific knowledge, particularly through genomics and evolutionary biology, provides new evidence of how ‘close’ vertebrate laboratory animals – including the mouse, the world's most common experimental vertebrate model – are to human beings. This evolutionary closeness holds enormous potential for biomedical science, even though it is not possible to easily transfer the results from animal studies to the human condition. However, this gain in knowledge also increased public attitudes that regard vertebrate animals as fellow creatures close to us humans. We already know that animals can not only feel pain and suffer but have considerable cognitive and emotional abilities too. These discoveries do not degrade *Homo sapiens*, but rather ‘upgrade’ the social status of mammals. In Germany, this ‘upgrading’ culminated in Article 20a of the German Constitution (https://www.bundestag.de/gg#) that gives animal protection a constitutional status. While this does not mean that animals have the same rights as humans, it recognizes them as living and feeling creatures worthy of recognition and protection.

During the past few decades, research has contributed significantly to raising awareness of animal welfare.

The social debate about the recognition of animals as fellow and valuable creatures is not only taking place in Germany: political movements to ban the use of animals in research and drug development, both from non‐governmental organizations and politics, have been gaining increasing influence worldwide. By way of example, in 2021 the European Parliament requested from the European Commission to develop an EU‐wide action plan to accelerate the phasing‐out of animal testing (https://www.europarl.europa.eu/news/en/press‐room/20210910IPR11926/meps‐demand‐eu‐action‐plan‐to‐end‐the‐use‐of‐animals‐in‐research‐and‐testing ). The Commission welcomed the resolution to discontinue animal testing in the long term and referred to the existing Directive 2010/63/EU, which acknowledges that animal testing is still necessary but has the same long‐term goal (https://oeil.secure.europarl.europa.eu/oeil/popups/summary.do?id=1675295&t=e&l=en).

This did not stop NGOs from launching the European Citizens' Initiative with the aim to end animal testing and which has been signed by more than one million people in the EU (https://www.hsi.org/news‐media/european‐citizens‐demand‐an‐end‐to‐animal‐testingeng/). In parallel, a fourth public initiative in Switzerland demands the end of all experiments on living creatures in the country (https://www.swissinfo.ch/eng/politics/swiss‐voters‐reject‐animal‐testing‐ban/47343764). Even though the Swiss voters overwhelmingly rejected previous initiatives for a ban on animal testing, the pressure still increases. Outside Europe, the US Environmental Protection Agency in Washington, DC, announced in 2019 that requests and funding of mammal studies will be reduced by 30 percent by 2025 and, if possible, aims to exclude any studies on mammals after 2035 (https://www.epa.gov/sites/default/files/2019‐09/documents/image2019‐09‐09‐231249.pdf). In Canada, the Minister of Health Mandate Letter signed in 2021 by Prime Minister Justin Trudeau states that a legislation shall be introduced to end testing on animals (https://pm.gc.ca/en/mandate‐letters/2021/12/16/minister‐health‐mandate‐letter). Australia implemented a ban on the use of animal test data for cosmetics from 2020 onward (https://www1.health.gov.au/internet/main/publishing.nsf/Content/ban‐cosmetic‐testing‐animals).

… political movements to ban the use of animals in research and drug development, both from non‐governmental organizations and politics, have been increasing influence worldwide.

These societal debates around the world follow and fuel dynamic social changes regarding the recognition and status of animals. As animal testing cannot be fully replaced by alternative methods, animal protection must be reconciled with the freedom of science and research. Nevertheless, science should continue to improve animal welfare and create more transparency about its use of animals before societal movements and politics lobby for more restrictive legal frameworks.

## Killing with reasonable cause

German laws on animal welfare do not allow pain, suffering, or harm to be inflicted on an animal without “reasonable cause”; this also applies to the use of animals in experiments and laboratory animals. This renders German animal welfare laws uniquely strict, especially regarding the criminal relevance of killing animals without a reasonable cause. Except for Austria, no other EU member state has included the reasonable cause in its legislation. The term itself, however, remains undefined in the German Animal Welfare Law and must be assessed on a case‐by‐case basis. Scientific research is a reasonable cause as the gain of knowledge outweighs the suffering of the animal if it cannot be achieved by alternative methods. However, a reasonable cause is not necessarily the case if surplus animals are killed without being used for scientific research.

… a reasonable cause is not necessarily the case if surplus animals are killed without being used for scientific research.

Agriculture is facing a similar conundrum for the poultry industry: worldwide, millions of male chicken are immediately killed after hatching because they cannot lay eggs, and it is not economical to feed them for meat production. The German Federal Administrative Court banned this practice in 2019 (https://www.bverwg.de/en/130619U3C28.16.0). In its judgment, the court argues that the life and well‐being of every animal has to be protected and that the cause to kill male chicks must be valid, rational, and supported by an interest that outweighs the interest of the animal in its integrity. Economic reasons alone are not sufficient for a reasonable cause. A legal consequence of the judgment is that violators may face prison for up to 3 years or a fine. Beyond Germany, France has also now banned the killing of day‐old male chicken, while the EU Commission has proposed an EU‐wide phase‐out of the practice (https://www.euractiv.com/section/agriculture‐food/news/commission‐to‐propose‐eu‐wide‐phaseout‐of‐male‐chick‐killing).

## Criminal charges against laboratory animal facilities

Encouraged by the ruling, German NGOs filed criminal complaints against 15 laboratory animal facilities accusing these of killing surplus laboratory animals without a reasonable cause (https://www.aerzte‐gegen‐tierversuche.de/de/news/aktuelle‐news/3577‐uni‐kiel‐toetet‐22‐497‐tiere‐als‐ueberschuss; https://www.aerzte‐gegen‐tierversuche.de/de/news/aktuelle‐news/3373‐ueber‐150‐000‐tiere‐als‐ueberschuss‐illegal‐getoetet). Two NGOs advocate the opinion that “surplus animals must either be conveyed or they must be cared for in special nursing homes for laboratory animals until the end of their natural lives” (https://www.aerzte‐gegen‐tierversuche.de/images/pdf/recht/ueberzaehlige_tiere_felde_kronaus.pdf).

[…] German NGOs filed criminal complaints against 15 laboratory animal facilities accusing these of killing surplus laboratory animals without a reasonable cause.

It is still not clear whether all criminal complaints will be prosecuted in court, but the large number of criminal charges has created uncertainty among the German research community (https://www.uni-muenster.de/news/view.php?cmdid=12097&lang=en). If court cases would indeed end in criminal convictions, the consequences for scientists and for biomedical research in Germany would be dramatic. German science organizations fear considerable restrictions in research with animals and disadvantages in international competitiveness (Feldwisch‐Drentrup, 2022). Keeping all surplus laboratory animals until their natural deaths would significantly hinder research and slow scientific progress (Chmielewska *et al*, 2015) as a large amount of funds and other resources would be needed to keep all these animals alive until their natural deaths.

The German Federal Institute for Risk Assessment (BfR) fulfills the role of the ‘National Committee for the Protection of Animals Used for Scientific Purposes’ – or National Committee – under the German Animal Welfare Act. This is not a unique German institution: The EU Directive 2010/63/EU requests every member state to establish such a National Committee to advise competent authorities and animal welfare bodies on the acquisition, breeding, housing, care, and use of animals in experiments and to ensure best practice. They further exchange information on animal welfare agencies and project evaluation within the EU.

The German National Committee first addressed legal questions on reasonable cause in 2015 (Chmielewska *et al*, 2015) when it assessed if feeding dead surplus animals like mice and rats to other animals, for instance, carnivores in zoos, can be a legitimate reason. For genetically altered animals, it would, however, require liberalizing the EU's restrictive regulations on GM food and feed (Hose *et al*, 2022). Using surplus animals as animal feed might be a reasonable cause, as their breeding and death serve a purpose. However, this new purpose of animal feed is not the original purpose – experimental usage – for their generation in breeding programs. A German Higher Administrative Court took a similar view of using male chicken for feed (https://www.justiz.nrw.de/nrwe/ovgs/ovg_nrw/j2016/20_A_488_15_Urteil_20160520.html) and did not consider the killing for feed as a purpose, but as a mere consequence.

## Surplus animals might emigrate

Poultry breeders may show scientists the way. Since the German ban on killing male chicken after hatching, an increasing number of German egg producers now import female chicken from abroad where the practice of killing male hatchlings is legal (https://www.euractiv.com/section/agriculture‐food/news/commission‐to‐propose‐eu‐wide‐phaseout‐of‐male‐chick‐killing). Thus, the strict German animal welfare laws have not solved the problem but merely outsourced it. The same could happen to animal‐based research: scientists would just move to countries with less restrictive animal welfare laws.

Research using non‐human primates could provide a glimpse into the future (Cyranoski, 2016; Chatfield & Morton, 2018). European scientists are increasingly collaborating with research institutions outside of the EU owing to high political and social pressure and the EU Directive 2010/63/EU on the protection of laboratory animals, which makes biomedical research with primates increasingly demanding (Hau *et al*, 2014; Chatfield & Morton, 2018). Many have moved experiments using monkeys to China (Cyranoski, 2016), where animal welfare regulations are less strict, either via collaborators or by setting up their own laboratories. The consequence is that *“*China is positioning itself as a world leader in primate research” (Cyranoski, 2016). The specific situation in Germany and the current uncertainty about the reasonable cause could induce even more scientists and companies to outsource their research.

… even with the best and most‐thought‐out breeding strategies and wide use of non‐animal alternatives, it is yet not possible to prevent surplus animals completely.

However, surplus animals are a global problem. The legal concept of reasonable cause may be specific to Germany, but the ethical dilemma crosses national borders. German scientists emigrating to conduct their research elsewhere will not reduce the overall number of surplus animals worldwide. It is an ethical challenge for science everywhere that has to be solved.

## First step ‐ reducing the emergence of surplus animals

Optimized breeding strategies can significantly reduce the number of surplus animals, and there are several guidance documents available (https://assets.publishing.service.gov.uk/government/uploads/system/uploads/attachment_data/file/773553/GAA_Framework_Oct_18.pdf; https://www.gv‐solas.de/wp‐content/uploads/2022/03/gen_2022_03_Reducing‐surplus‐experimental‐animal‐generation‐1.pdf). An interview with Sara Wells, Director of the Mary Lyon Centre in the UK, explains how current advances in breeding and genotyping, as well as the use of gene editing tools, are leading to a refinement and reduction in the use of laboratory animals (https://www.criver.com/eureka/between‐3rs‐do‐new‐gene‐editing‐tools‐mean‐fewer‐animals). In particular, she discusses the use of genetically controlled inbred lines for breeding; redefinition of colony management: cryopreservation, blastocyst and sperm genotyping; and gene editing, and how these could help create more refined mouse lines that, for example, reflect human diseases more accurately.

CRISPR/Cas9 gene editing could also help reduce the number of surplus animals with the undesired sex. Douglas *et al* (2021) demonstrated an embryonically lethal CRISPR/Cas9 strategy that produces male‐ or female‐only litters with 100 % efficiency.

The need to maintain a large number of animals for experiments can also be reduced in the future as more complex, non‐animal models are becoming increasingly available. In his recent article entitled “Is it Time for Reviewer 3 to Request Human Organ Chip Experiments Instead of Animal Validation Studies?” Ingber (2020) discusses the advances of organoids and microphysiological systems.

There are other alternatives available such as the use of invertebrate model organisms. Owing to advanced genetics and gene editing tools, the well‐established model organisms *Caenorhabditis elegans* and *Drosophila melanogaster* can now be used to mimic human disease functions (Baldridge *et al*, 2021). In particular, their short generation time, the large pool of knowledge and genetics tools, and publicly available strains would make it easier for researchers to study disease function or respond to reviewers' request without relying on vertebrate models (Baldridge *et al*, 2021).

Nonetheless, even with the best and most‐thought‐out breeding strategies and wide use of non‐animal alternatives, it is yet not possible to prevent surplus animals completely. Hence, additional strategies for their (ethical) usage are needed.

## Female animals are not of the wrong sex

Another option to reduce the number of surplus animals is to use both sexes in animal experiments; this would just follow the NIH Policy on sex as a biological variable (https://orwh.od.nih.gov/sites/orwh/files/docs/NOT‐OD‐15‐102%20Guidance.pdf). It would actually “kill two birds with one stone”, as the usage of both sexes increases the quality of research and reduces the number of unused animals.

Science already provides the arguments to do so. Male laboratory animals still dominate biomedical research because many scientists believe that female animals are too variable for routine use in experiments (Beery & Zucker, 2011). This bias is also one reason for the lack of translatability and reproducibility in preclinical research (Zucker & Beery, 2010). For that reason, NIH Policy requires that “strong justification from the scientific literature, preliminary data, or other relevant considerations must be provided for applications proposing to study only one sex” (https://orwh.od.nih.gov/sex‐gender/nih‐policy‐sex‐biological‐variable). Implementing this policy might help reduce the number of surplus animals not only at the institutional level but also among commercial breeders. However, not all researchers are willing to follow this appeal by the NIH (Waltz *et al*, 2021).

## Transparency is needed

Despite all the established and ongoing measures to reduce the number of surplus animals, the societal pressure is still on. EU parliamentarians are calling for an EU action plan to totally eliminate the use of animals in research and drug testing, emphasizing the development of alternative methods. Not without reason, as the EU Directive 2010/63/EU obliges scientists to develop said alternative methods. Consequently, supporting this development would result in fewer surplus animals.

In 2022, the EU member states have counted their surplus animals and will report their numbers to the EU commission this year; publication is expected for 2024. This will be an important moment as it will make surplus animals visible again. Society does accept that animal testing is still necessary for medical progress and to fight diseases. However, the public must know about the costs of animal testing too: animal suffering eases human suffering. We have to live with this reality, but we should try everything we can do to remedy this moral dilemma and promote the development of new alternative methods. The ethical handling of surplus animals has the power to accelerate animal welfare.

## Disclosure and competing interests statement

All authors have no conflict of interest. All authors are employed at the German Federal Institute for Risk Assessment and part of the German Centre for the Protection of Laboratory Animals (Bf3R). Gilbert Schönfelder is also employed at Charité—Universitätsmedizin Berlin, corporate member of Freie Universität Berlin and Humboldt‐Universität zu Berlin.

## Supporting information


