# Supplementary material for: The fate of surplus laboratory animals: Minimizing the production of surplus animals has greatest potential to reduce the number of laboratory animals
Source: EMBO Rep. 2023 Jan 30;24(3):e56551. doi: 10.15252/embr.202256551 (PMC9986809; doi:10.15252/embr.202256551)
Supplement: Supplementary file 1 [file EMBR-24-e56551-s001.pdf]

EMBO reports

The fate of surplus laboratory animals

Hartmut Wewetzer, Tobias Wagenknecht, Bettina Bert and Gilbert Schönfelder
DOI: 10.15252/embr.202256551

Corresponding author(s): Gilbert Schönfelder (gilbert.schoenfelder@bfr.bund.de) , Hartmut Wewetzer (hartmut.wewetzer@bfr.bund.de)

Review Timeline:

Submission Date:	24th Nov 22
Editorial Decision:	28th Nov 22
Revision Received:	19th Dec 22
Accepted:	17th Jan 23

Editor: Holger Breithaupt

Transaction Report: This article was editorially reviewed.
